# Efficacy of Phosphatidylinositol-3 Kinase Inhibitors in a Primary Mouse Model of Undifferentiated Pleomorphic Sarcoma

**DOI:** 10.1155/2012/680708

**Published:** 2012-04-29

**Authors:** Suzy Kim, Rebecca D. Dodd, Jeffrey K. Mito, Yan Ma, Yongbaek Kim, Richard F. Riedel, David G. Kirsch

**Affiliations:** ^1^Department of Radiation Oncology, Duke University Medical Center, Box 91006, Durham, NC 27710, USA; ^2^Department of Radiation Oncology, Boramae Hospital, Seoul National University Hospital, Seoul 156-707, Republic of Korea; ^3^Department of Pharmacology and Cancer Biology, Duke University Medical Center, Box 91006, Durham, NC 27710, USA; ^4^Department of Clinical Pathology, College of Veterinary Medicine, Seoul National University, Seoul 156-707, Republic of Korea; ^5^Division of Medical Oncology, Duke University Medical Center, Box 91006, Durham, NC 27710, USA

## Abstract

Recent advances in sarcoma genomics have identified novel mutations in the PI3K pathway in human sarcomas. Here, we use a mouse model of primary soft-tissue sarcoma for preclinical testing of doxorubicin and inhibitors of the PI3K pathway: BKM120 (PI3K inhibitor) and BEZ235 (a dual PI3K/mTOR inhibitor). Doxorubicin-treated tumors (*n* = 15) showed a partial response rate of 6.6%, just as the majority of human sarcomas do not respond to doxorubicin. Treatment with BKM120 elicited a partial response in 50% of tumors (*n* = 10), which was also seen in combination with doxorubicin (*n* = 10). Additionally, BKM120 treatment produced a robust delay in tumor growth kinetics. BEZ235-treated tumors (*n* = 9) showed a complete response rate of 11.1%. Combining BEZ235 with doxorubicin (*n* = 10) increased the complete response rate to 50% (*P* = 0.035). These studies demonstrate that PI3K pathway inhibition is a viable and attractive target for soft-tissue sarcomas.

## 1. Introduction

Sarcomas are a rare, heterogeneous group of mesenchymal neoplasms. Systemic chemotherapeutic options are limited both in number and efficacy for patients with advanced disease. Doxorubicin is currently one of the most commonly used chemotherapeutic agents for patients [1]. Meta-analysis of eight randomized, multicenter studies [2] has estimated response rates of 17–27% for single-agent doxorubicin (60–80 mg/m^2^). However, there is a severe risk of cardiac toxicity associated with high cumulative doses of doxorubicin, in addition to other side effects including nausea, anemia, and compromised immune function. Novel tumor-specific targets for chemotherapies would increase the available options for sarcoma treatment and perhaps reduce the potential side effect profile associated with current treatment approaches.

 Genomic analyses of sarcomas with complex karyotypes have recently identified novel mutations that may be targeted by molecularly directed therapies [3]. One of the most frequent somatic mutations is found in the PIK3CA gene, which encodes the catalytic subunit of phosphatidylinositol-3 kinase (PI3K). The PI3K pathway is commonly involved in epithelial malignancies but has not been fully explored as a target for sarcoma therapy. Initial studies showed that the PI3K/mTOR inhibitor BEZ235 inhibited growth of Ewing's sarcoma and rhabdomyosarcoma xenografts [4]. However, to the best of our knowledge, neither PI3K inhibitors nor standard-of-care chemotherapies have been investigated in a genetically engineered mouse model of soft-tissue sarcoma. In this study, we test the efficacy of doxorubicin and two PI3K inhibitors (BKM120 and BEZ235) in an immunocompetent model of temporally—and spatially—restricted soft-tissue sarcoma.

 We have previously utilized the Cre-loxP system in mice to activate conditional mutations in *Kras* and *Trp53* (LSL-Kras^G12D^; p53^flox/flox^) by intramuscular delivery of Cre recombinase to generate high-grade primary soft-tissue sarcomas [5]. Cross-species genomic analysis revealed that this tumor model most closely resembles human undifferentiated pleomorphic sarcoma (UPS) [6]. Because this model is temporally—and spatially—restricted, tumor size and response to treatment can be easily monitored. Here, we use this model to test the response of primary mouse sarcomas to doxorubicin and to inhibition of the PI3K pathway with BKM120 (PI3K inhibitor) and BEZ235 (dual inhibitor of PI3K and mTOR), both provided by Novartis. Activation of the PI3K pathway by growth signals initiates a signaling cascade resulting in phosphorylation of AKT, activation of mTOR, and subsequent phosphorylation of the ribosomal subunit S6 [7]. Therefore, the juxtaposition of the pan-PI3K inhibitor (BKM120) and the PI3K/mTOR dual inhibitor (BEZ235) may reveal important information about the design of future molecularly targeted therapies for soft-tissue sarcomas.

## 2. Results

### 2.1. Inhibition of PI3K and mTOR in BEZ235- and BKM120-Treated Mouse Sarcoma Cell Lines

**Figure 1:**
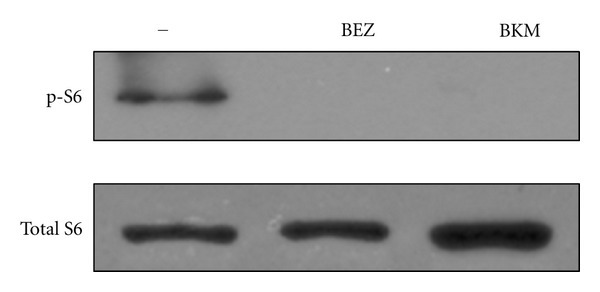
Mouse sarcoma cell line 4515 treated with BEZ235 and BKM120. Cells were treated with 500 nM BEZ235 or 500 nm BKM120 for 18 hours. Western blot shows levels of total-S6 and phospho-S6.

Before embarking on *in vivo* screening of the compounds, we first tested the agents *in vitro* with cells derived from primary sarcomas in LSL-Kras^G12D^; p53^flox/flox^ mice. The sarcoma cells were treated with either the dual PI3K/mTOR inhibitor BEZ235 or the PI3K inhibitor BKM120 for 18 hours prior to harvest. The BEZ235-treated cells exhibited decreased levels of phospho-S6, a molecule downstream of the mTOR pathway (Figure 1). Additionally, the BKM120-treated cells demonstrated decreased levels of phospho-S6, showing the PI3K pathway was inhibited through inhibition of this downstream target. These data suggest that BEZ235 treatment in mouse sarcoma cells effectively inhibits targets of the mTOR pathway and BKM120 treatment results in inhibition of PI3K pathway targets.

### 2.2. Doxorubicin Treatment of Primary Sarcomas in Mice

**Figure 2:**
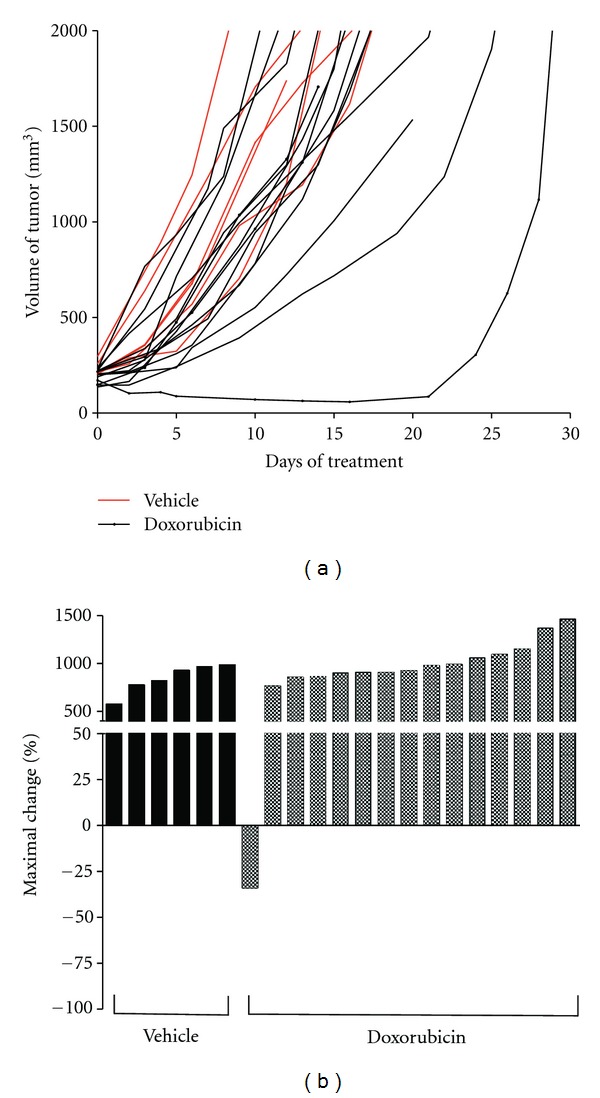
(a) Tumor volume of mice treated with vehicle alone (red) or doxorubicin alone (black). (b) Waterfall plot of tumor volume response for vehicle (black bars) and doxorubicin (shaded bars). Data is presented as percent maximal loss in tumor volume for animals responding to treatment and as percent maximal gain in tumor volume for nonresponders.

Doxorubicin is the most common standard-of-care chemotherapy for advanced stage sarcoma [1]. To model doxorubicin therapy in a genetically engineered mouse model of sarcoma, we used Cre-loxP technology to generate primary high-grade soft-tissue sarcomas in mice. Tumors were generated by intramuscular delivery of an adenovirus that expresses Cre recombinase into compound mutant mice with conditional mutations in both oncogenic *Kras* (LSL-Kras^G12D^) and mutant *Trp53* (p53^flox/flox^) [5]. Approximately 2-3 months later, these mice developed tumors resembling human UPS/MFH (malignant fibrous histiocytoma) at the site of injection [6]. Doxorubicin treatment was started when the tumor volume was between 150 and 300 mm^3^, and tumor volumes were monitored, on average, twice weekly. Data are presented both as absolute tumor volume (Figure 2(a)) and in a waterfall plot that displays maximal percent change in volume for each tumor following treatment (Figure 2(b)). For the waterfall plots, tumors that responded to treatment are shown as the greatest percent *loss* in tumor volume and tumors with a maximal change of −100% represent tumors that fully regressed and achieved a complete response. Tumors that did not respond to treatment are shown as the greatest percent *gain* in tumor volume during treatment. Animals treated with vehicle alone showed no tumor response (*n* = 0/6). In mice receiving an initial dose of doxorubicin (10 mg/kg), the growth of several tumors was delayed, although only 6.6% of tumors showed partial response to treatment (*n* = 1/15). This is similar to human sarcomas, where the majority of patients with sarcomas do not show a response to treatment with doxorubicin [2].

### 2.3. BKM120 Treatment of Primary Sarcomas in Mice

**Figure 3:**
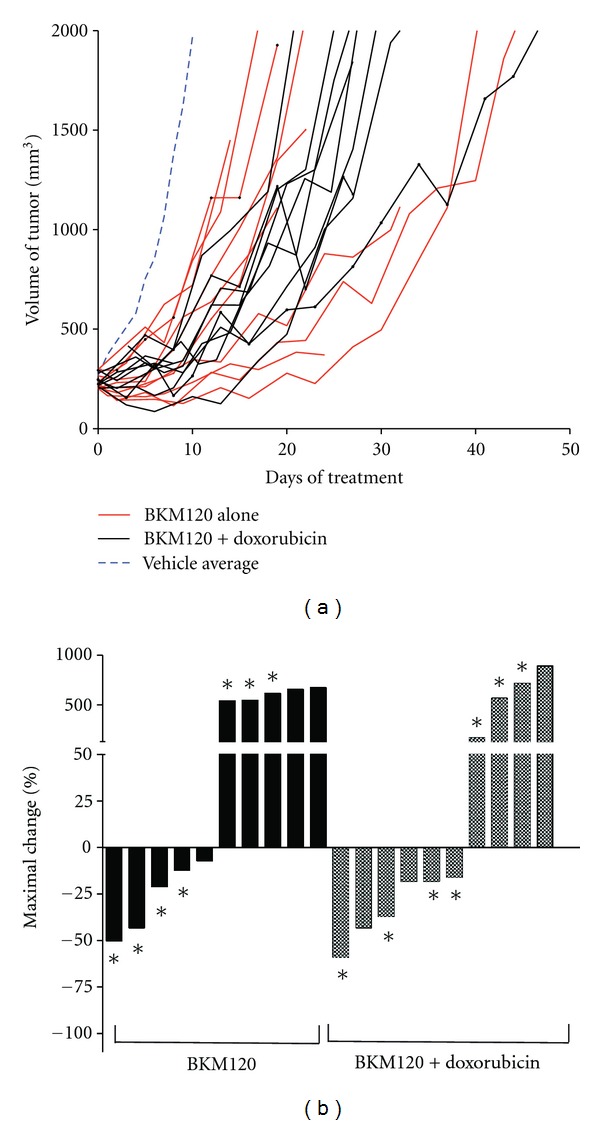
(a) Tumor volume of mice treated with BKM120 alone (red) or BKM120+doxorubicin (black). The average growth of vehicle-treated tumors (blue) from Figure 2 is shown for comparison. (b) Waterfall plot of tumor volume response for BKM120 (black bars) and BKM120+doxorubicin (shaded bars). Data is presented as percent maximal loss in tumor volume for animals responding to treatment and as percent maximal gain in tumor volume for nonresponders. Tumors that began treatment below 250 mm^3^ are marked with an asterisk (∗).

To test the effect of inhibiting the PI3K pathway in primary sarcomas *in vivo*, we used the novel PI3K inhibitor, BKM120. Mice with tumors 150–300 mm^3^ in volume were treated with BKM120 (50 mg/kg, administered 5 days/week) for 4–7 weeks. Treatment with either BKM120 or BKM120 with doxorubicin delayed tumor growth in comparison to vehicle alone (Figure 3, Table 1). Fifty percent of tumors treated with BKM120 alone (*n* = 5/10) showed a partial tumor response. The combination of BKM120 with doxorubicin had a partial response rate of 60% (*n* = 6/10), which was similar to BKM120 single-agent treatment (*P* = 0.343). Response rates were not improved by beginning treatment on tumors at volume less than 250 mm^3^ instead of greater than 250 mm^3^ (*P* = 0.076) (Table 2).

**Figure 4:**
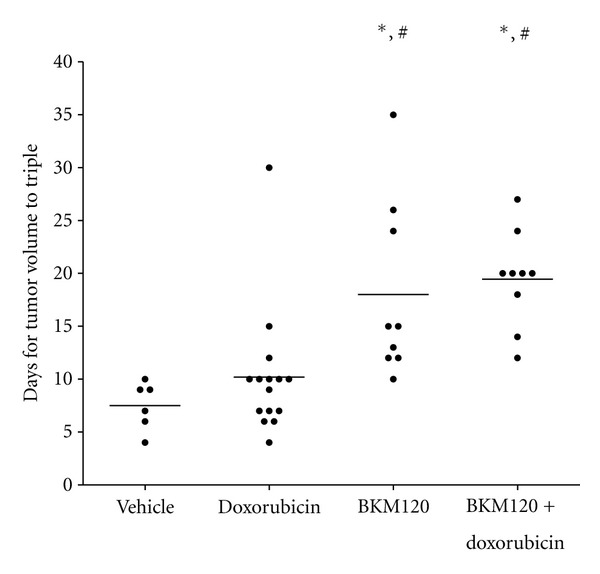
Time required for BKM120-treated tumors to triple in volume. A paired Student's *t*-test was performed. Groups statistically different from vehicle alone are marked with an # (*P* < 0.05), and groups statistically different from doxorubicin alone are marked with an ∗ (*P* < 0.05).

 In addition to observing tumor response to treatment, the time required for a tumor to triple in volume was calculated (Figure 4). This measurement can determine differences in growth kinetics between groups that achieved partial response to treatment [8]. Vehicle-treated tumors tripled in size with an average 7.5 days (range 4–10 days), whereas doxorubicin-treated tumors required an average of 10.2 days (range 6–30 days) to triple in volume (*P* = 0.31). Tumors treated with BKM120 required 18.0 days (range 10–35 days) to triple in volume (*P* = 0.0133, compared to vehicle alone). The combination of BKM120 with doxorubicin had the greatest time to triple the tumor volume of 19.4 days (range 12–27 days), but this was not statistically different than BKM120 alone (*P* = 0.657).

### 2.4. BEZ235 Treatment of Primary Sarcomas in Mice

**Figure 5:**
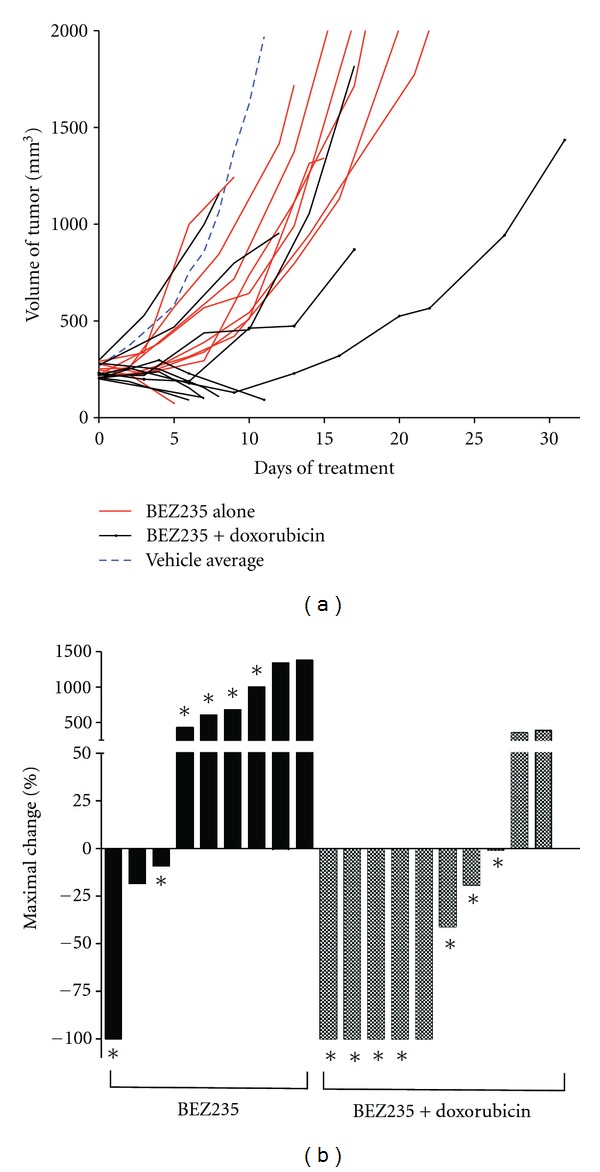
(a) Tumor volume of mice treated with BEZ235 alone (red) or BEZ235+doxorubicin (black). The average growth of vehicle-treated tumors (blue) from Figure 2 is shown for comparison. (b) Waterfall plot of tumor volume response for BEZ235 (black bars) and BEZ235+doxorubicin (shaded bars). Data is presented as percent maximal loss in tumor volume for animals responding to treatment and as percent maximal gain in tumor volume for nonresponders. Tumors that began treatment below 250 mm^3^ are marked with an asterisk (∗).

 To test the effect of inhibiting both the PI3K and mTOR pathways simultaneously with the BEZ235 inhibitor, tumor-bearing mice were treated with BEZ235 alone (50 mg/kg, administered 5 days/week) or a combination of doxorubicin and BEZ235. Drug treatment began when tumor volumes reached 150–300 mm^3^ and continued for 3-4 weeks. Tumor volume was measured twice weekly, on average. Tumor growth was delayed in mice receiving BEZ235 or BEZ235 with doxorubicin in comparison to vehicle alone (Figure 5, Table 3). Complete tumor response was observed in 11.1% of the BEZ235-treated mice (*n* = 1/9), and 22.2% of tumors responded partially to treatment (*n* = 2/9). Interestingly, combining BEZ235 with doxorubicin resulted in a 50% complete response rate (*n* = 5/10), much higher than that observed for either treatment alone (*P* = 0.035). Additionally, 30% of tumors treated with the BEZ235 and doxorubicin combination showed partial response (*n* = 3/10). In contrast to results with BKM120, response rates were improved when tumors were treated at volume of less than 250 mm^3^ compared to a volume greater than 250 mm^3^ (*P* = 0.025) (Table 4).

### 2.5. Toxicity of Treatment

During the experiments with BEZ235, some mice died 6–8 days after drug administration. To assess toxicity of BEZ235 or BKM120, we measured the weight of mice with tumors treated with doxorubicin, BKM120, or BKM120 and doxorubicin. We also assessed weight loss in non-tumor-bearing, wild-type 129/SvJae mice treated with BEZ235 and doxorubicin for 5 days per week for two weeks. We recorded the percent change in body weight twice weekly with a loss greater than 15% considered a terminal endpoint (Supplementary Figure 1 in supplementary Material available on line at doi:10.1155/2012/680708). With this criterion, no toxicity was observed with doxorubicin treatment alone (Table 5). In contrast, animals receiving the combined BEZ235 and doxorubicin regimen showed significant weight loss, with 50% of animals losing >10% of their body weight. BKM120-treated animals did not exhibit such severe weight loss. Twenty percent of BKM120-treated animals lost 10–15% of body weight, and no animal lost greater than 15% of initial weight. Similar results were found when BKM120 was combined with doxorubicin, with only 1 of 9 animals losing >10% of body weight.

## 3. Materials and Methods

### 3.1. Mouse Sarcoma Model

All mouse work was performed in accordance with Duke University Institutional Animal Care and Use Committee approved protocols. Tumors were generated by injection of Adenovirus-expressing Cre recombinase (University of Iowa, Vector core) into the lower left leg of p53^flox/flox^; LSL-Kras^G12D^ compound mutant mice as previously described [5]. After 2-3 months, tumors were detected in the lower limb of animals. Tumor volumes were calculated using the formula *V* = (*π* × *L* × *W* × *H*)/6, with *L*, *W*, and *H* representing the length, width, and height of the tumor in mm, respectively.

### 3.2. Doxorubicin and PI3K Inhibitor Treatment

Treatment started when tumors were between 150 and 300 mm^3^. Mice receiving vehicle alone were treated with NMP 10% (1-methyl-2-pyrrolidone)/PEG300 90% (Fluka) orally 5 days per week. Doxorubicin (Sigma) was dissolved in 2% DMSO in PBS and injected intraperitoneally as a single bolus at 10 mg/kg once on the first day of the treatment. BEZ235 (Novartis) or BKM120 (Novartis) was dissolved in NMP 10% (1-methyl-2-pyrrolidone)/PEG300 90%. Mice were treated with 50 mg/kg of BEZ235 [4, 22] or BKM120 orally 5 days per week from the first day of the treatment. Treatment for all mice continued until tumors regressed or it was necessary to sacrifice the mouse for animal welfare concerns (i.e., animal was moribund; tumor volume reached 2000 mm^3^; animal lost >15% body weight).

### 3.3. Tumor Growth Analysis

Tumor response is defined as any tumor volume that is less than baseline. A complete tumor response is defined as complete regression of a tumor so that it is no longer palpable. For waterfall plot analyses, percent maximal change is reported as the greatest percent loss in tumor volume from baseline for tumors responding to treatment. A value of −100% represents tumors that completely responded to treatment and were no longer detectable. Tumors that did not respond to treatment are shown as the greatest percent gain in tumor volume from baseline over the course of the treatment. Time required for tumors to triple in volume was determined by reporting the day on which a tumor volume reading exceeded three times the initial volume [8]. Mice excluded from this analysis included animals that were sacrificed before the tumor could triple in volume (1 BKM120-alone mouse and 1 BKM120/doxorubicin mouse).

### 3.4. Statistical Analysis

Because the primary tumors were treated across a span of several months, it was important to verify that tumors were measured an equal number of times across all treatment groups. Tumors were measured an average of 3.0 times during days 1–10 and an average of 2.2 times during days 11–20. For days 1–10, and the vehicle-alone, doxorubicin-alone, BKM120-alone, BKM120+doxorubicin, BEZ235-alone, and BEZ2235+doxorubicin cohorts were measured an average of 3.0, 2.9, 3.1, 3.2, 2.9, and 2.7 times, respectively. For days 11–20, the vehicle-alone, doxorubicin-alone, BKM120-alone, BKM120+doxorubicin, BEZ235-alone, BEZ2235+doxorubicin cohorts were measured an average of 2.2, 2.3, 2.6, 2.7, 2.2, and 1.0 times, respectively. None of these measurement intervals were statistically different, with the exception of the BEZ235+doxorubicin tumors during days 11–20 (*P* < 0.001), which resulted from the loss of mice during this time window. Graphs and statistics were performed in Graph Pad 4.0. A paired, 2-tailed Student's *t*-test was performed to determine differences between treatment groups.

### 3.5. Toxicity Studies

Healthy, wild-type 129S4/SvJae mice were treated with BEZ235+doxorubicin for 5 days per week for two weeks. Percent change in body weight was recorded twice weekly, with a loss greater than 15% considered a terminal endpoint. Tumor-bearing mice receiving treatment with doxorubicin alone, BKM120 alone, or BKM120+doxorubicin were also monitored for changes in body weight.

### 3.6. Western Blot Analysis

The mouse sarcoma cell line 4515 was derived from a primary sarcoma in a LSL-Kras^G12D^; p53^flox/flox^ mouse. The 4515 cells were cultured in DMEM + 10% FBS. They were treated with either BKM120 or BEZ235 (500 nM) for 18 hours prior to harvest. Cells were washed once with cold PBS (Sigma) and lysed for 10 minutes on ice with RIPA buffer (Sigma), supplemented with phosphatase inhibitors (Sigma, P5726 and P0044). Protein concentration was measured with BCA Protein Concentration Assay (Thermo Scientific). MiniProtean TGX gels (BioRad) were transferred to PVDF by wet transfer. Antibodies were from Cell Signaling, including total S6 (no. 9202) and phospho-S6 (no. 5364).

## 4. Discussion

 We have utilized a genetically engineered mouse model of soft-tissue sarcoma to perform preclinical studies of doxorubicin and PI3K pathway inhibitors. To our knowledge, this is the first study to use PI3K inhibitors and standard-of-care chemotherapies in a primary mouse model of soft-tissue sarcoma. We have assessed endpoints that are similar to those used in human clinical trials, including complete and partial response rates. We observed that treatment with standard-of-care doxorubicin produced a low response rate in a genetically engineered mouse model of sarcoma. As the majority of human sarcomas do not respond to doxorubicin, these results help credential the LSL-Kras^G12D^; p53^flox/flox^ mouse model for testing novel therapeutic agents. Treatment with the PI3K inhibitor BKM120 showed a partial response as a single agent and provided tumor-stabilizing effects along with greatly slowed tumor growth. This suggests that inhibition of PI3K alone is sufficient to see a robust delay in tumor growth. Combining BKM120 with doxorubicin increased benefits, although these did not reach statistical significance. The average time to tumor tripling for BKM120 and doxorubicin was 1.4 days more than BKM120 alone, which is similar to the 1.2-day increase for doxorubicin alone compared to vehicle. This suggests that any interaction between BKM120 and doxorubicin is additive. However, it is important to note that using the protocol of doxorubicin administration in this study, neither the benefits of doxorubicin alone compared to vehicle nor doxorubicin addition to BKM120 treatment was statistically different. Treatment with the dual PI3K/mTOR inhibitor BEZ235 produced a minimal tumor response that increased dramatically when BEZ235 was combined with doxorubicin. However, severe toxicity was observed in half of the animals given the combined BEZ235/doxorubicin treatment. Of note, BKM120 alone or BKM120/doxorubicin treatment was better tolerated than BEZ235. The complete response observed with BEZ235, particularly when combined with doxorubicin, suggests that combining PI3K and mTOR inhibition may be a promising therapy, but care must be taken to address safety and toxicity concerns. Taken together, these promising pre-clinical results suggest that research exploring the potential benefits of PI3K inhibition in sarcoma patients is warranted.

 Genetically engineered mouse models are vital tools for cancer drug development [9], and they possess several advantages over traditionally used xenograft models. First, these models recapitulate tumor-stroma interactions that are fundamental to tumor growth and treatment response. This point is especially salient when considering the contribution of microenvironmental factors to overall tumor evolution, including vasculature and immune cells. Second, the additional heterogeneity found in primary tumors in comparison to single-cell derived xenografts better models the genetic diversity of human tumors that may influence response to treatments. Indeed, several genetically engineered mouse models are better predictors of therapeutic outcomes than xenograft models [10]. For example, addition of erlotinib to conventional chemotherapy showed no improvement in progression-free survival for both a large patient cohort and a Kras-mutant NSCLC mouse model; in contrast, xenograft models suggested that erlotinib would increase efficacy of treatments in Kras-mutant NSCLC cell lines [10]. Third, mouse models allow for studies that would be complex or impossible to conduct during patient trials, such as stopping responsive therapy to determine tumor control, testing combination therapy for agents that are only in phase I development, or modeling therapeutic resistance.

 Results from studies in genetically engineered mouse models can be further translated into clinical parameters by calculating endpoints that are similar to those used in human clinical trials. Reporting values such as overall survival (OS) and progression free survival (PFS) [10] for mouse studies can allow direct comparison of data with large patient cohorts. This information is important to determine if mouse models respond similarly to standard-of-care chemotherapies before they are used as platforms for screening novel therapeutic agents. In the current study, we chose to report the additional information of complete response rate and partial response rate to treatment, in an attempt to extend these observations towards clinical relevance. Indeed, this measurement allows comparison of the low response rates to doxorubicin treatment in both this mouse study and patient reports. Observing tumor characteristics that are predictive of outcomes in patient situations, such as tumor size at treatment initiation, is another way that mouse studies can inform patient treatment. An interesting observation from our study was the striking difference in tumor response based on initial size of the treated tumors with BEZ235. This volumetric response of tumors suggests this therapy may be beneficial in the adjuvant setting to treat minimal residual disease. Additionally, our results suggest that clinical trials for BEZ235 should evaluate whether tumor burden at the beginning of treatment influences treatment response.

 Although drug development in genetically engineered mouse models is advantageous, there are several limitations to these studies. Primarily, these models are unable to reflect the vast genetic diversity found in human tumors. The mutational heterogeneity of patient disease is one of the main reasons clinical trials do not show the promising results predicted from laboratory tests. Genotype-directed clinical trials may improve upon these results. The LSL-Kras^G12D^; p53^flox/flox^ sarcomas are driven by overactive Kras, which may increase sensitivity to PI3K inhibition. Therefore, these results may only be applicable to patients with mutations in PI3K-sensitive pathways.

 PI3K inhibitors have shown efficacy against a wide array of cancers in a laboratory setting. BEZ235 decreased cell proliferation of breast cancer cell lines with PIK3CA mutations [11] and cisplatin-resistant human ovarian cancer cells [12]. Additionally, BEZ235 suppressed growth of xenograft models of breast cancer [11], rhabdomyosarcoma [4], metastatic melanoma [13], gastric cancer [14], hepatocellular carcinoma [15], renal cell carcinoma [16], prostate cancer [17, 18], primary effusion lymphoma [19], glioma [18, 20], and non-small-cell lung cancer [21]. BEZ235 has also shown activity in genetically engineered mouse models, including Kras-initiated ovarian carcinoma [12]. Alternatively, single-agent BEZ235 therapy was not effective in a primary NSCLC mouse model, but combining BEZ235 with a MEK inhibitor did inhibit tumor growth [22]. This suggests that PI3K-directed drugs may be most effective when combined with other targeted therapies.

 Molecularly targeted therapy against the PI3K pathway is rapidly advancing into clinical use. BEZ235 is currently in phase I/II clinical trials in patients with advanced solid tumors, including advanced breast cancer. In this study, patients are prescreened for molecular alterations in PIK3CA and/or PTEN. Such genotype-directed clinical trials may determine if genetic mutations in the PI3K pathway contribute to treatment response. According to http://clinicaltrials.gov/, new BEZ235 trials are opening for patients with advanced endometrial cancer and metastatic breast cancer. Several Phase II trials for BKM120 are currently recruiting patients with recurrent glioblastoma, metastatic renal cell carcinoma, metastatic castration-resistant prostate cancer, metastatic non-small-cell lung cancer, and HER2-positive breast cancer. Additional phase 1 studies are using alternative PI3K inhibitors in combination with other agents, such as standard-of-care chemotherapies, MEK inhibitors, or Bevacizumab. We believe that the data presented in the paper suggests that PI3K-directed therapy may be beneficial for sarcoma patients and should be further explored.

## Supplementary Material

To assess potential toxicity following drug treatment, mice were monitored for changes in body weight. Tumor-bearing mice were treated with doxorubicin alone (A), BKM120 alone (C) or BKM120 *+* doxorubicin (D). Healthy, wild-type 129/SvJae mice were treated with BEZ235 *+* doxorubicin (B) for two weeks to monitor toxicity.

## Figures and Tables

**Table 1:** Response rates of BKM120-treated tumors.

	Tumor response rate		
	Complete	Partial	All responders
BKM120	0/10	5/10	5/10 (50%)
BKM120+Dox	0/10	6/10	6/10 (60%)

Total	0/20	11/20	11/20 (55%)

**Table 2:** Response rates of BKM120-treated tumors stratified by initial tumor volume.

	Initial tumor volume		*P* < 0.05	Total

	<250 mm^3^	>250 mm^3^
BKM120 tx: complete responders	0/14	0/6	no	0/20 (0%)
BKM120 tx: all responders	8/14	3/6	no	11/20 (55%)

**Table 3:** Response rates of BEZ235-treated tumors.

	Tumor response rate		
	Complete	Partial	All responders
BEZ235	1/9	2/9	3/9 (33%)
BEZ235+Dox	5/10	3/10	8/10 (80%)

Total	6/19	5/19	11/19 (58%)

**Table 4:** Response rates of BEZ235-treated tumors stratified by initial tumor volume.

	Initial tumor volume		*P* < 0.05	Total

	<250 mm^3^	>250 mm^3^
BEZ235 tx: complete responders	5/13	1/6	yes	6/19 (32%)
BEZ235 tx: all responders	9/13	2/6	yes	11/19 (58%)

**Table 5:** Toxicity of BEZ235 and BKM120 scored by percent loss in body weight.

	Number of mice with maximal loss in body weight			
	5–10%	>10%	# >15%	Total
Doxorubicin alone	0/8 (0%)	0/8 (0%)	0	0/8 (0%)
BEZ235+Dox	2/10 (20%)	5/10 (50%)	2	7/10 (70%)
BKM120 alone	3/10 (30%)	2/10 (20%)	0	5/10 (50%)
BKM120+Dox	2/9 (22%)	1/9 (11%)	1	3/9 (33%)
