# Supplementary material for: Efficacy of Phosphatidylinositol-3 Kinase Inhibitors in a Primary Mouse Model of Undifferentiated Pleomorphic Sarcoma
Source: Sarcoma. 2012 Apr 29;2012:680708. doi: 10.1155/2012/680708 (PMC3350993; doi:10.1155/2012/680708)
Supplement: Supplementary file 1 — To assess potential toxicity following drug treatment, mice were monitored for changes in body weight. Tumor-bearing mice were treated with doxorubicin alone (A), BKM120 alone (C) or BKM120 + doxorubicin (D). Healthy, wild-type 129/SvJae mice were treated with BEZ235 + doxorubicin (B) for two weeks to monitor toxicity. [file 680708.f1.pdf]

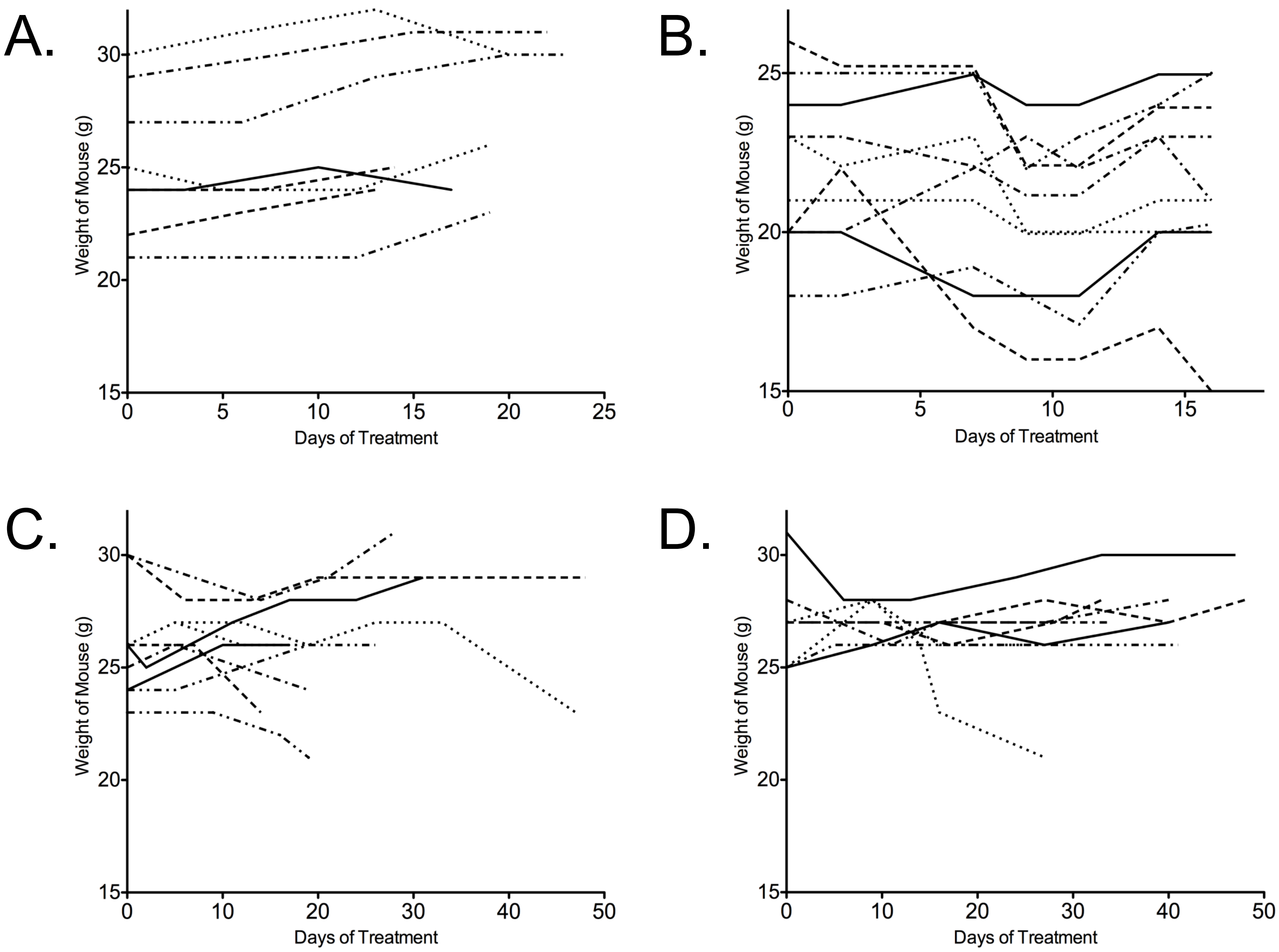

Supplemental Figure 1. Changes in body weight for mice treated with (A) doxorubicin alone, (B) BEZ235 + doxorubicin, (C) BKM120 alone, and (D) BKM120 + doxorubicin.
